# Insect chaperones Hsp70 and Hsp90 cooperatively enhance toxicity of *Bacillus thuringiensis* Cry1A toxins and counteract insect resistance

**DOI:** 10.3389/fimmu.2023.1151943

**Published:** 2023-04-20

**Authors:** Blanca Ines García-Gomez, Tamara Alejandrina Sánchez, Sayra Natalia Cano, Nathaly Alexandre do Nascimento, Alejandra Bravo, Mario Soberón

**Affiliations:** Departamento de Microbiología Molecular, Instituto de Biotecnología, Universidad Nacional Autónoma de México, Cuernavaca, Morelos, Mexico

**Keywords:** heat shock proteins, chaperones, Bacillus thuringiensis, cry toxins, protoxins, toxin receptors, Insect resistance

## Abstract

*Bacillus thuringiensis* (Bt) produces different insecticidal proteins effective for pest control. Among them, Cry insecticidal proteins have been used in transgenic plants for the control of insect pests. However, evolution of resistance by insects endangers this technology. Previous work showed that the lepidopteran insect *Plutella xylostella* PxHsp90 chaperone enhanced the toxicity of Bt Cry1A protoxins by protecting them from degradation by the larval gut proteases and by enhancing binding of the protoxin to its receptors present in larval midgut cells. In this work, we show that PxHsp70 chaperone also protects Cry1Ab protoxin from gut proteases degradation, enhancing Cry1Ab toxicity. We also show that both PxHsp70 and PxHsp90 chaperones act cooperatively, increasing toxicity and the binding of Cry1Ab439D mutant, affected in binding to midgut receptors, to cadherin receptor. Also, insect chaperones recovered toxicity of Cry1Ac protein to a Cry1Ac-highly resistant *P. xylostella* population, NO-QAGE, that has a disruptive mutation in an ABCC2 transporter linked to Cry1Ac resistance. These data show that Bt hijacked an important cellular function for enhancing its infection capability, making use of insect cellular chaperones for enhancing Cry toxicity and for lowering the evolution of insect resistance to these toxins.

## 1 Introduction

*Bacillus thuringiensis* (Bt) produce diverse insecticidal proteins that have been used in the control of insect pests in agriculture (1, 2). The most successful application of Bt insecticidal proteins has been their expression in transgenic crops, showing efficient pest control with an important reduction on the use of chemical insecticides (3). Among the different insecticidal proteins produced by Bt, the Cry proteins are the most commercially used. These proteins bind to different membrane gut proteins known as Cry-receptors such as aminopeptidase N, alkaline phosphatase, cadherin or ABC transporters (4). However, the successful application of Cry toxins in insect pest control, has been challenged by the evolution of resistance to Cry toxin action in different insect pests, where high levels of resistance have been shown to be linked to mutations affecting the expression of Cry receptors (5, 6). Besides the resistance problem, there are some important crop pests that show low susceptibility to the Cry toxins that are currently used in transgenic plants (7), thus it is important to improve Cry toxin action against these particular insect pests.

Cry proteins are synthesized as protoxins and show specific toxicity to the insect larval stages. The Cry1 protoxins are composed of seven structural domains that are activated by the larval gut proteases leading to the formation of the three-domain toxin core. Domain I is involved in toxin oligomerization and pore formation, while domains II and III are involved in the insect specificity by their specific binding to different membrane receptors inducing toxin oligomerization, insertion into the cell membrane and pore formation, causing larval gut cells-burst by osmotic shock (4, 8). Recent data have shown that Cry protoxins also bind to some Cry-receptors before being activated by gut proteases, inducing a robust pore formation activity in the midgut cells (9, 10). It was reported that the oligomers formed from protoxin or from activated toxin differ in their sensitivity to heat, membrane insertion capabilities and their pore characteristics (9). Therefore, it has been proposed that Cry toxins have a dual mode of action, one for the protoxin and another for the activated toxin (10, 11). Several insects that have evolved resistance to the activated Cry1Ac toxin, were still susceptible to the protoxin, supporting a dual mode of action (11).

Proteomic analysis indicated that, besides binding of Cry proteins to larval membrane gut proteins, Cry toxins can also bind to some intracellular proteins such as actin, V-ATP synthase beta subunit and the Hsp70 chaperone among others (12, 13). Since Cry proteins exert their toxicity in the plasma membrane by forming lytic pores, the potential role of all these intracellular proteins in the mechanism of action of Cry proteins has not been studied in detail. Recently, it was shown that *Plutella xylostella* Hsp90 chaperone enhanced the toxicity of Cry1Ab and Cry1Ac toxins against different lepidopteran pests (14). The enhancing effect of Hsp90 on Cry1A toxicity was shown to be due to the protection of the protoxin degradation by gut proteases and also by improving protoxin binding to the cadherin receptor (14). Interestingly, *P. xylostella* Hsp70 also enhanced Cry1Ac toxicity, in contrast to a bacterium GroEL chaperone that showed marginal effect (14). It was shown that insect Hsp70 or Hsp90 chaperones resist degradation by the larval gut proteases, in contrast to GroEL that is highly sensitive to degradation, explaining the efficacy of Hsp70 or Hsp90 in enhancing Cry toxicity when fed to the larvae (14).

Hsp70 is recognized as an Hsp90 co-chaperone. Hsp70 assist unfolded intermediates by recognizing stretches of hydrophobic residues, and re folding these intermediates to their native state, while Hsp90 assist later stages in the folding or activation of these proteins (15, 16). Here we show that besides the independent enhancement of Cry1Ab toxicity performed by both chaperones, the PxHsp70 and PxHsp90 chaperones act together in assisting Cry1A protoxins for receptor binding. In addition, both PxHsp70 and PxHsp90 act cooperatively assisting Cry1Ac toxicity and countering the insect resistance to Cry proteins that is linked to mutations affecting ABCC2 receptor. These results suggest that the use of insect chaperones along with Cry toxins could be useful for the control of insect pests that show low susceptibility to these toxins and also to counteract the Cry toxin resistance that has evolved in different insect pests.

## 2 Materials and methods

### 2.1 Expression of Cry1Ab, Cry1AbR99E, Cry1AbE129K, Cry1AbG439D and Cry1Ac

To produce Cry1A protoxins, the acrystalliferous Bt407 *cry* ^-^ strain transformed with the corresponding plasmids (pHT315-Cry1Ab, pHT315-Cry1AbR99E, pHT315-Cry1AbE129K, pHT315-Cry1AbG439D) or the HD73 strain were grown in sporulation medium plates until complete sporulation (17). For the growth of Cry1Ab or Cry1Ab mutants this medium was supplemented with erythromycin 10 μg/ml. The spore/crystals were harvested and washed three times with distilled water and finally suspended in solubilization buffer (50 mM Na_2_CO_3_ pH 10.5, 0.02% β-mercaptoethanol) and incubated for 1 h at 37°C. The soluble protoxins were separated from non-soluble spores and debris by centrifugation, the protein concentration of the soluble material was determined by Bradford method using BSA as standard protein and the quality of soluble protoxin samples was analyzed in 12% acrylamide SDS-PAGE electrophoresis.

### 2.2 Production of PxHsp70, PxHsp90 and cadherin fragment CR12

For the production of PxHsp70 or PxHsp90 chaperones, the genes of these proteins were cloned in pET28b expression vector and their production was done in *E. coli* BL21cells as reported (14). Briefly, 2.5 ml of overnight cultures of BL21-PxHsp90 and BL21-PxHsp70 strains in 2x-TY medium supplemented with 50 μg/ml kanamycin were used to inoculate 250 ml of the same medium and incubated at 37°C with 200 rpm until an OD_600_ of 0.6 was reached. After this, the expression of PxHsp90 or PxHsp70 was induced by adding 0.5 mM isopropyl β-D-1-thiogalactopyranoside (IPTG) to the cultures and incubating overnight at 30°C with 150 rpm shaking. Cells were harvested by centrifugation, frozen and suspended in PBS containing 8 M Urea. Then the samples were lyzed by sonication four times for 50 sec at 4°C and centrifuged 40 min at 48,000 rpm. The supernatant was loaded into Ni-NTA agarose column, washed with 10 volumes of 35 mM imidazole in PBS and eluted with 250 mM imidazole in PBS supplemented with 1 mM ATP and 1 mM MgCl_2_. The chaperone samples were finally dialyzed by using Amicon Ultra 30K filters into PBS buffer supplemented with 1 mM ATP, 1 mM MgCl_2_. These chaperone samples were used immediately or stored at 4°C for using them the next day.

Cloning of the cadherin fragment CR12 into pET22 vector was previously reported (18). The pET22-CR12 plasmid was transformed into *E. coli* ER2566 strain (Invitrogen, Carlsbad CA) and cells were grown at 37°C in TY medium supplemented with 100 mg/ml ampicillin and 0.1% glucose until they reached an OD of 0.7 at 600 nm. The expression of CR12 fragment was induced with 1 mM IPTG and grown for additional 4 h at 25°C. Cells were harvested by centrifugation, frozen and suspended in PBS containing 8 M Urea. The samples were lysed by sonication four times, for 50 sec at 4°C and centrifuged 40 min at 48,000 rpm. The CR12 peptide protein was purified through a nickel affinity column and eluted with 250 mM imidazole in PBS as described above for purification of the chaperone proteins.

### 2.3 Analysis of Cry1Ab protoxin stability to proteolysis in the presence of PxHsp70

For the analysis of the effect of PxHsp70 on the stability of Cry1Ab protoxin to protease treatment, 150 μg of PxHsp70 or BSA were incubated with 2.5 μg Cry1Ab protoxin in PBS buffer supplemented with 1 mM ATP and 1mM MgCl_2_ in a final volume of 50 μl. Trypsin, (60 ng in 2 μl) was added and incubated at 37°C with agitation at 850 rpm. Ten μl samples were taken after different incubation times (3, 5, 10, 20 or 60 min) mixed with 3 μl of 4X-Laemmli buffer and boiled 3 min. Time 0 corresponds to the time that takes to add trypsin to the mixture, remove a 10 μl sample and boil it for 3 min Laemmli buffer to stop the reaction. All other time samples were removed at the indicated times and treated similarly to time 0 sample. These samples were loaded in SDS/PAGE 10% acrylamide gel and transferred into PVDF membrane (Millipore) for western blot detection.

In the case of the treatment with midgut juice from *P. xylostella*, the midgut juice was prepared from midgut tissues dissected from 4^th^ instar larvae as previously described (14). Briefly, twenty dissected midguts were paced in an Eppendorf tube and centrifuged at 2200 *xg* for one min. The supernatant was collected and protein concentration was determined by Bradford assay using BSA as standard. A total of 150 μg PxHsp70 or BSA were incubated with 2.5 μg Cry1Ab protoxin in PBS buffer supplemented with 1 mM ATP and 1 mM MgCl_2_ in a final volume of 50 μl. Fifty ng of midgut juice protein in 2 μl, were added and incubated at 37°C with 850 rpm agitation. Ten μl samples were taken after different incubation times (30, 60, 90, or 120 min) mixed with 3 μl of 4X-Laemmli buffer and boiled for 3 min. Time 0 corresponds to the time that takes to add the midgut juice protein to the mixture, remove a 10 μl sample and stop the reaction by boiling 3 min in the Laemmli buffer. Samples were loaded in SDS/PAGE 10% acrylamide gel and transferred into PVDF membrane (Millipore) for western blot detection. The PVDF membranes were blocked with 3% non-fat dried skimmed milk in PBS. Then, membranes were incubated 1 h at room temperature with anti-Cry1Ab antibody (1:20,000 dilution) in PBS. After washing three times for 15 min with washing buffer (0.05% tween in PBS) the membrane was incubated with a HRP conjugated anti-IgG rabbit antibody (Sigma) (1:30,000 dilution) for 1 h at room temperature and washed three times with washing buffer. SuperSignal™ chemiluminescence (Pierce) was used for signal detection according to manufacturer´s instructions.

### 2.4 Binding of PxHsp70 to Cry1Ab protoxins

Binding of Hsp70 to Cry1Ab, Cry1AbR99E, Cry1AbE129K or Cry1AbG439D was analyzed by ELISA binding assays. ELISA 96 well plates were coated with 0.5 μg of the different Cry proteins suspended in 50 μl of PBS, by overnight incubation at 4°C. Unbound protoxins were washed away with PBS buffer and the plate was blocked with 2% Slim milk in PBS for 2 h at 37°C. The blocking buffer was removed and 50 μl of Hsp70 solution (250 or 500 nM) were added to the wells and incubated 1 h at 37°C. The ELISA plates were washed three times with PBS buffer and then incubated with 50 μl anti-polyHistidine-Peroxidase conjugated antibody (1:5,000 dilution) (Sigma-Aldrich) for 1 h a 30°C. Finally, 50 μl of 1 mg/ml *o*-phenylenediamine in substrate buffer (100 mM K_3_PO_4_, pH 5) supplemented with 2 μl of hydrogen peroxide was added to each well and the reaction was stopped by adding 25 μl of 6 N HCl. The plates were read on microtiter plate reader at OD 490 nm. Data represent means of triplicates and each experiment was repeated twice. The *p* value was calculated by using a student’s *t* -test for two groups (https://www.graphpad.com/quickcalcs/ttest1/), *p* values < 0.05 were considered as statistically significant differences.

### 2.5 Binding analysis of Cry1Ab protoxins to cadherin fragment CR12 in the presence of PxHsp70 and PxHsp90

ELISA plates were coated with 0.5 µg of *M. sexta* cadherin fragment (CR12) in 100 µl of PBS per well over night at 4°C. Plates were washed three times with PBS and then blocked with 200 µl/well of PBS-M (PBS, 2% skim milk) for 2 h at 37°C. After blocking, the plates were washed three times with PBS and incubate 1 h at 37°C with different concentrations of Cry1Ab protoxins (25, 50 or 100 nM) in the presence or absence of 30-fold higher PxHsp70 concentration, previously incubated with the protoxin during 10 min at 30°C in a total 100 µl volume of PBST (PBS + 0.1% Tween 20). Wells were then washed three times with PBS. The bound proteins were finally detected by using anti-Cry1Ab polyclonal antibody (1: 20,000 dilution) for 1 h at 37°C in 100 µl PBST buffer. Blots were washed three times with PBST and three times with PBS, and then incubated with 100 µl of PBST containing anti-rabbit HRP conjugated antibody (1: 20,000 dilution) (Santa Cruz Biotechnology) for 1 h at 37°C. Finally, the reaction was developed with HRP substrates *o*-phenylenediamine and hydrogen peroxide as described above. Each experiment was performed in duplicate with three repetitions. The statistical calculations (mean and standard deviation) and graphics were performed using the Microsoft Excel Program. The *p* value was calculated by using a student’s *t* -test for two groups (https://www.graphpad.com/quickcalcs/ttest1/), *p* values < 0.05 were considered as statistically significant differences.

### 2.6 Analysis of Cry1Ab or Cry1Ac toxicity in the presence of PxHsp70 and PxHsp90

Bioassays were performed using 24 wells plates containing one larvae per well. Third instar larvae of *P. xylostella* were used. Larval diet was surface contaminated with different concentrations of Cry1Ab or Cry1Ab mutants soluble protoxins as stated in each experiment plus different concentrations of chaperone. Controls with the same protoxins concentration without chaperone addition were included. For the bioassays against *P. xylostella* NO-QAGE strain, the larval diet was surface contaminated with 25 ng/cm^2^ of Cry1Ac soluble protoxin plus different concentrations of chaperone, and the controls were incubated with Cry1Ac protoxin alone without chaperone. The soluble protoxin was previously incubated with the mixture of chaperones for 30 min at 37°C in 1mM MgCl_2_, 1mM ATP, in PBS buffer before contamination of the larval diet. Mortality was assessed after 7 days. Each experiment was performed in triplicate (72 larvae per treatment). As negative controls, the diet was surface contaminated with the highest chaperone concentration or water. Data are shown in mean ± SD. The *p* value was calculated by using a student’s *t* -test for two groups (https://www.graphpad.com/quickcalcs/ttest1/), *p* values < 0.05 were considered as statistically significant differences.

## 3 Results

### 3.1 PxHsp70 protects Cry1Ab protoxin from midgut proteases

**Figure 1:**
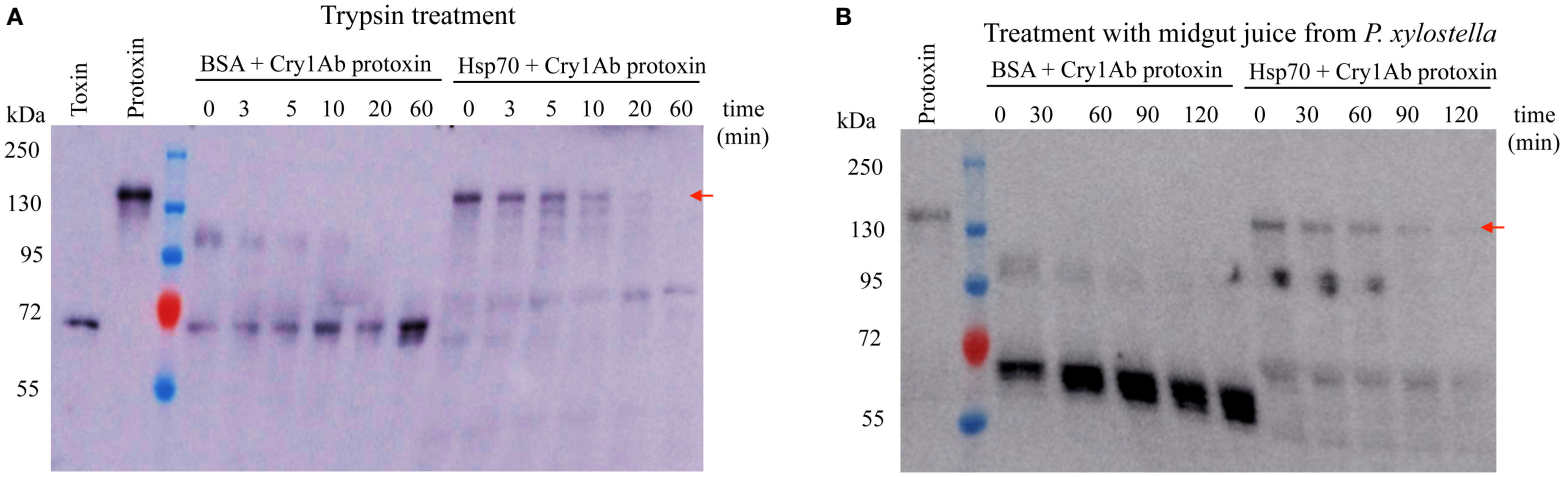
PxHsp70 protects Cry1Ab protoxin from protease degradation. **(A)** Trypsin treatment (1:40 w:w) of one μg Cry1Ab protoxin for different time points in the presence of either BSA or PxHsp70 and revealed by western blot using anti-Cry1Ab antibody. **(B)** Treatment of Cry1Ab protoxin with midgut juice from *P. xylostella* as described in methods. One μg Cry1Ab protoxin was treated with the midgut proteases for different time points in the presence of either BSA or PxHsp70 and revealed by western blot using anti-Cry1Ab antibody. Representative results of three replicas are shown. The arrows indicate the position of the 130 kDa Cry1Ab protoxin.

Previously we showed that PxHsp70 enhanced Cry1Ac toxicity against *Plutella xylostella* in a dose dependent way (14). In addition, Cry1Ab protoxin was shown to be protected by PxHsp90 from degradation by *P. xylostella* midgut proteases which correlated with the enhancing effect of PxHsp90 on Cry1Ab toxicity (14). To determine if PxHsp70 also has a similar protection effect of Cry1Ab protoxin against midgut proteases, we analyzed the stability of Cry1Ab protoxin after trypsin treatment, which is routinely used for *in vitro* activation of Cry toxins, or after treatment with *P. xylostella* midgut juice in the presence or absence of PxHsp70. In the control without PxHsp70, BSA was added at the same concentration as PxHsp70 in order to discard a non-specific protein protecting effect against protease treatment. **Figure 1A** shows that in the presence of PxHsp70, Cry1Ab protoxin was protected from trypsin degradation since the 130 kDa protoxin band was still observed after 10 min of trypsin treatment in contrast to the control samples without PxHsp70, where the protoxin band was not observed at all incubation times, indicating a fast degradation under these conditions. A similar result was obtained when Cry1Ab protoxin was incubated with *P. xylostella* midgut juice since the 130 kDa band was still observed after 90 min incubation with midgut juice in the presence of PxHsp70 in contrast with the control samples where Cry1Ab 130 kDa protoxin band was not observed, suggesting immediate degradation by the midgut juice proteases (**Figure 1B**).

### 3.2 PxHsp70 and PxHsp90 cooperatively assist Cry1Ab in receptor binding

**Figure 2:**
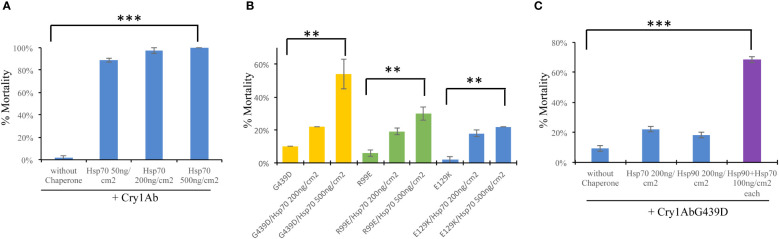
PxHsp70 and PxHsp90 recovers toxicity of Cry1AbG439D against *Plutella xylostella*. **(A)** Effect of PxHsp70 on Cry1Ab toxicity. Percentage of mortality (%) of *P. xylostella* larvae treated with 2.5 ng/cm^2^ of Cry1Ab in the presence of 50, 200 or 500 ng/cm^2^ of PxHsp70 chaperon (***p> 0.0001). **(B)** Effect of PxHsp70 on the toxicity of non-toxic Cry1Ab mutants affected in different steps of the mode of action. Percentage of mortality (%) of *P. xylostella* larvae treated with 25 ng/cm^2^ of Cry1AbG439D (affected in receptor binding), Cry1AbR99E (affected in oligomerization) or Cry1AbE129K (affected in oligomer membrane insertion) assayedin the presence of different concentrations of PxHsp70 (200 and 500 ng/cm^2^). *P* values indicated statistically significant differences: **p> 0.0024 with Cry1AbG439D; **p> 0.0016 with Cry1AbR99E, and **p> 0.0006 with Cry1AbE129K mutant. **(C)** Effect of both chaperones, PxHsp70 and PxHsp90, on the toxicity of Cry1AbG439D mutant (affected in receptor binding). Percentage of mortality (%) of *P. xylostella* larvae treated with 25 ng/cm^2^ of Cry1AbG439D, mutant toxin in the presence of different concentrations of PxHsp70 (200 ng/cm^2^), PxHsp90 (200 ng/cm^2^) or a mixture of both chaperones (100 ng/cm^2^ of each chaperone) (*P* values indicated statistically significant differences: ***p<0.0001). Data with standard deviations represent means of three treatments using 24 larvae per treatment in each repetition.

An interesting feature of Hsp90 chaperone is its capacity to suppress mutations of its client proteins (19, 20). Previously, we showed that PxHsp90 preferentially recovered the toxicity of Cry1AbG439D mutant affected in receptor binding (21) in contrast to the toxicity of Cry1AbR99E or Cry1AbE129K mutants affected on oligomerization or oligomer membrane insertion respectively (22, 23), indicating that Hsp90 assist Cry1Ab binding to midgut receptors (14). To determine what step of the mode of action of Cry1Ab protein is assisted by PxHsp70, the toxicity of the three non-toxic Cry1Ab mutants described above was analyzed in the presence of PxHsp70 against *P. xylostella* larvae. First, **Figure 2A** shows that PxHsp70 (50 ng/cm^2^, 200 ng/cm^2^ or 500 ng/cm^2^) enhanced the toxicity of Cry1Ab (2.5 ng/cm^2^) from 3% mortality to 100% mortality (*p*> 0.0001) as was previously reported (14). Toxicity assays of 25 ng/cm^2^ from each Cry1Ab mutant performed in the presence of 200 ng/cm^2^ or 500 ng/cm^2^ of PxHsp70 showed that Cry1AbG439D recover substantial toxicity (from 10% up to 54% mortality in the presence of 500 ng/cm^2^ of PxHsp70; *p*<0.0024) (**Figure 2B**) in contrast of the Cry1AbR99E (from 5% mortality to 30% mortality in the presence of 500 ng/cm^2^ of PxHsp70; *p*<0.0016) or Cry1AbE129K (2% mortality up to 22% mortality with 500 ng/cm2 PxHsp70; *p*<0.0006) mutants that showed a subtle increase in toxicity in the same experimental conditions (**Figure 2B**), suggesting that PxHsp70 assists Cry1Ab binding to midgut receptors.

**Figure 3:**
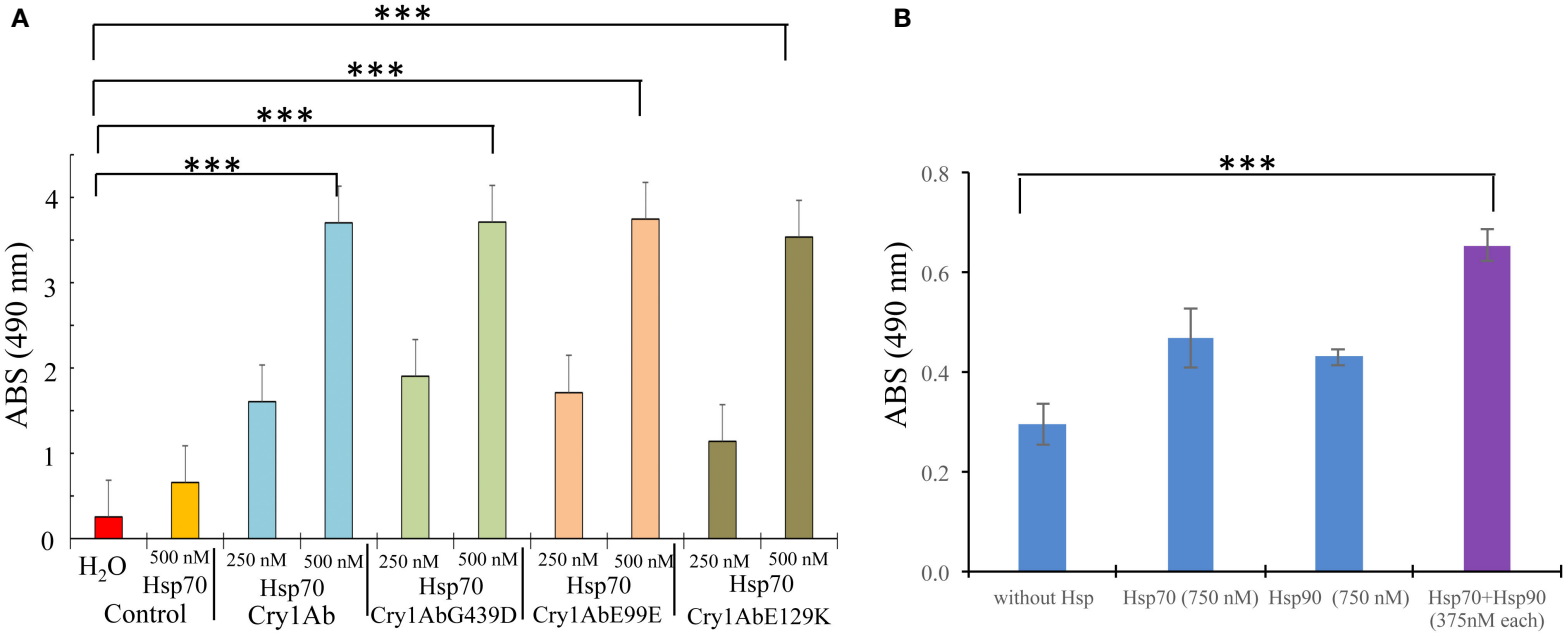
PxHsp70 and PxHsp90 assist Cry1Ab protoxin in binding to CAD receptor fragment. **(A)** ELISA binding assays of Cry1Ab, Cry1AbG439D, Cry1AbR99E or Cry1AbE129K protoxins to PxHsp70. ELISA plates were coated with 0.5 μg of each protoxin and after washing unbound proteins, the plates were incubated with different concentrations of PxHsp70, and revealed with anti-His antibody as described in methods. *P* values indicated statistically significant differences: ***p> 0.0001. **(B)** ELISA binding assays of Cry1AbG439D protoxin (100 nM) and the mixtures of Cry1AbG439D protoxin (100 nM):PxHsp70 (750 nM), Cry1AbG439D protoxin (100 nM):PxHsp90 (750 nM) or Cry1AbG439D protoxin (100nM):PxHsp70/PxHsp90 (375 nM/375 nM)) to CAD CR12. After washing, the toxin bound to the CR12 was revealed with anti-Cry1Ab antibody as described in methods. *P* values indicated statistically significant differences: ***p> 0.0002.

To further analyze the effect of PxHsp70 on Cry1AbG439D protoxin, the binding of Cry1AbG439D protoxin to a *M. sexta* cadherin fragment CR12 was analyzed in the presence or absence of PxHsp70 by ELISA binding assays. This CR12 fragment contains a Cry1Ab binding site (18). **Figure 3A** shows that PxHsp70 bound similarly to Cry1Ab, Cry1AbR99E, Cry1AbE129K or Cry1AbG439D (p> 0.0001). **Figure 3B** shows that Cry1AbG439D (100 nM) did not recover substantial binding to CR12 in the presence of PxHsp70 (750 nM) or PxHsp90 (750 nM) under these experimental conditions (**Figure 3B**). However, in the presence of both chaperones, PxHsp70 and PxHsp90 (375 nM of each chaperone), the Cry1AbG439D mutant recover significant binding to CR12, higher than that observed with PxHsp70 or PxHsp90 alone (*p*<0.0002) (**Figure 3B**), suggesting that these two chaperones act cooperatively in assisting the binding of Cry1Ab to the CR12 receptor. Since both PxHsp70 and PxHsp90 act cooperatively in enhancing binding of Cry1AbG439D protoxin to the CR12 cadherin fragment, we analyzed the effect of both chaperons in the toxicity of Cry1AbG439D to *P. xylostella* larvae. **Figure 2C** shows that in the presence of both chaperons (100 ng/cm^2^ of each chaperone) Cry1AbG439D recover higher toxicity (from 9% mortality to 68% mortality; *p*<0.0001) compared with the effect observed with PxHsp70 (from 9% mortality to 22% mortality with 200 ng/cm^2^; *p*<0.0005) or PxHsp90 (from 9% mortality to 18% mortality with 200 ng/cm^2^; *p*<0.0001). These results show that both Hsp70 and Hsp90 act cooperatively to recover binding of Cry1AbG439D mutant to CR12 and to increase toxicity of this Cry1AbG439D mutant against *P. xylostella* larvae.

### 3.4 Hsp70 and Hsp90 act cooperatively in countering resistance to Cry1Ac insecticidal protein

**Figure 4:**
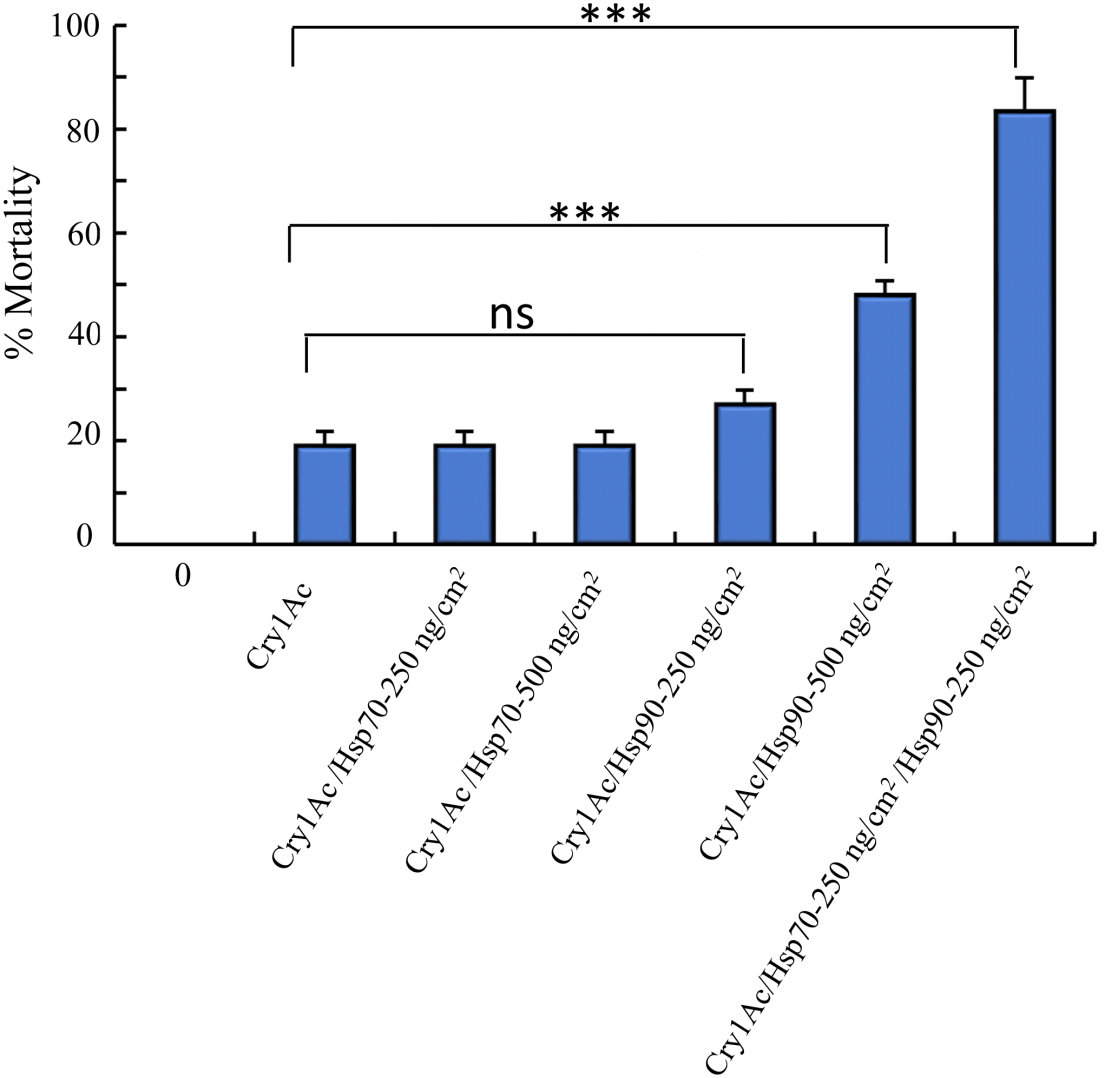
PxHsp70 and PxHsp90 recovers Cry1Ac toxicity against *Plutella xylostella* NO-QAGE Cry1Ac resistant strain. Percentage of mortality of Cry1Ac protoxin (25 ng/cm^2^) against *P. xylostella* NO-QAGE strain in the presence of different concentrations of PxHsp70 (250 or 500 ng/cm^2^), PxHsp90 (250 or 500 ng/cm^2^) or Pxhsp70+PxHsp90 (250 + 250 ng/cm^2^). Data with standard deviations represent means of three treatments using 24 larvae per treatment in each repetition. *P* values indicated statistically significant differences: ***p> 0.0001; ns, not significant difference.

High resistance to Cry toxins in different insects has been linked to mutations affecting the expression of Cry toxins receptors (6). It was previously described that the resistance of NO-QAGE *P. xylostella* population, that is highly resistant to Cry1Ac toxin (> 1000-fold resistance), was linked to a 30 bp deletion on the *ABCC2* gene, a functional Cry1Ac receptor (24). Therefore, we analyzed if PxHsp70 and PxHsp90 could counteract resistance to Cry1Ac toxin in the NO-QAGE strain. The 3^rd^ instar larvae of NO-QAGE strain were fed with Cry1Ac (25 ng/cm^2^) along with 250 or 500 ng of PxHsp70 or PxHsp90 or the mixture of the two chaperones (250 ng/cm^2^ of each chaperone). **Figure 4** shows that 500 ng/cm^2^ PxHsp70 had no significant effect on the Cry1Ac mortality to the NO-QAGE strain (19% mortality of Cry1Ac with or without PxHsp70), in contrast to 500 ng/cm^2^ PxPHsp90 that showed significant increase in mortality of Cry1Ac (from 19% mortality to 48% mortality; *p*< 0.0001). Finally, when 250 ng/cm^2^ of both chaperones were fed together along with the Cry1Ac toxin to the NO-QAGE strain a significant increase in mortality was observed (from 19% mortality to 84% mortality in the presence of both chaperones; *p*<0.0001). These results show that PxHsp70 and PxHsp90 act cooperatively to counteract insect resistance to Cry toxins (**Figure 4**).

## 4 Discussion

Bt hijacked an important cellular function for enhancing its infection capability, making use of insect cellular chaperones for enhancing Cry toxicity and for lowering the evolution of insect resistance to Cry toxins. In the case of different virus, it has been shown that Hsp90 assists certain viral proteins at different stages of infection (25), while in the case of the cholera toxin, Hsp90 assists the translocation of the catalytic subunit to the cytoplasm where it affects cAMP cellular levels (26) indicating that different microbial pathogens make use of cellular chaperones for their own benefit.

Previous work showed that that lowering down Hsp90 expression by RNAi in the mosquito *Aedes aegypti* resulted in a tolerant phenotype to Cry11Aa toxin (27). This result suggested that Hsp90 was somehow involved in the toxicity pathway of Cry toxins. Regarding the mechanism involved on the enhancement of Cry toxicity induced by Hsp90 action, previous work showed that PxHsp90 assists Cry1Ab or Cry1Ac toxins by protecting these protoxins from degradation by larval midgut proteases and also by enhancing binding of Cry1Ab to cadherin receptor (14). Here we show that PxHsp70 also protected Cry1Ab protoxin from degradation by larval gut proteases. However, PxHsp70 had no effect on binding of Cry1AbG439D to a CAD CR12 fragment receptor. We showed here that both chaperones act together since the binding of Cry1AbG439D to a cadherin fragment CR12 was much higher when both chaperones were used together with the Cry1AbG439D mutant in contrast when the mutant protein was treated with only PxHsp70 or PxHsp90. As described previously Hsp70 is consider a co-chaperone of Hsp90 since Hsp70 assist unfolded intermediates by recognizing stretches of hydrophobic residues to fold them into their native state, while Hsp90 assist later stages of folding or activation (15, 16). Thus, it is possible that the alkaline gut conditions of the larvae, partially unfold the Cry protoxins enhancing their degradation by gut proteases and limiting their binding to larval brush border membrane proteins.

Since PxHsp70 and PxHsp90 act cooperatively to recover the toxicity of Cry1AbG439D affected in receptor binding, we explored the possibility that these chaperones could recover the toxicity of Cry1Ac to a *P. xylostella* Cry1Ac resistant strain, NO-QAGE, whose resistance has been shown to be linked to a mutation in the *ABCC2* receptor gene (24). Here we show that both chaperones, PxHsp70 and PxHsp90 act cooperatively to recover the toxicity of Cry1Ac to the NO-QAGE strain. This result suggests that the insect chaperones assist Cry1Ac toxin to bind to other midgut proteins, besides ABCC2, recovering its toxicity. Although we have not identified the gut proteins involved in binding of Cry1Ac in the NO-QAGE strain in the presence of both chaperones it is possible that ABCC3 could be involved, since it has been shown that ABCC2 and ABCC3, orthologous proteins, have redundant roles in Cry1Ac toxicity in *P. xylostella* (28). Another possibility is that increased binding to cadherin may be involved, since we have shown that both PxHsp90 or PxHsp70 enhances binding of Cry1Ab protoxin to *M. sexta* cadherin (**Figure 3B**) (**14**).

Both Hsp70 and Hsp90 are intracellular proteins that have important roles in maintaining the cellular proteome homeostasis (15, 16). However, Cry toxins exert their toxicity by forming pores in the cellular membrane. It was recently shown that the mosquitocidal Cry11Aa toxin is internalized inside the midgut cells, although it is not clear if its internalization is related to toxicity (29). Since PxHsp70 and PxHsp90 protect Cry protoxins from gut proteases and also enhance binding to membrane gut proteins it is likely that their effect on Cry toxicity is exerted outside the cell. Cry toxins burst insect cells by osmotic shock and both Hsp70 and Hsp90 are abundant cellular proteins (15, 16, 30), thus it is possible that the insect chaperones are released, along with other proteins, into the gut lumen when cells are burst by osmotic shock, facilitating their interaction with the Cry toxins outside the cells. Bacterial infection can enhance chaperones expression as a result of cell stress (31). Thus, Bt took advantage of stress responses in arthropods to enhance its toxicity and to counter resistance to the Bt Cry toxins. In any case, the effect of insect chaperones in increasing Cry toxicity in susceptible and in Cry-resistant insects could have important biotechnological applications.

## Data availability statement

The original contributions presented in the study are included in the article/supplementary material. Further inquiries can be directed to the corresponding author.

## Author contributions

BG-G performed toxicity assays against Px NO-QAGE strain and binding analysis to CAD receptor, TS performed toxicity assays of Cry1Ab and Cry1Ab mutants and binding of Hsp70 to Cry1Ab mutants. SC performed analysis of Cry1Ab protoxin stability in the presence of Hsp70. NN prepare recombinant proteins from *E. coli*. AB planned experiments, prepare figures and wrote the manuscript. MS planned experiments, prepare figures and wrote the manuscript. All authors contributed to the article and approved the submitted version.
